# Interactions between WUSCHEL- and CYC2-like Transcription Factors in Regulating the Development of Reproductive Organs in *Chrysanthemum morifolium*

**DOI:** 10.3390/ijms20061276

**Published:** 2019-03-14

**Authors:** Yi Yang, Ming Sun, Cunquan Yuan, Yu Han, Tangchun Zheng, Tangren Cheng, Jia Wang, Qixiang Zhang

**Affiliations:** 1 Beijing Key Laboratory of Ornamental Plants Germplasm Innovation & Molecular Breeding, National Engineering Research Center for Floriculture, Beijing Laboratory of Urban and Rural Ecological Environment, Engineering Research Center of Landscape Environment of Ministry of Education, Key Laboratory of Genetics and Breeding in Forest Trees and Ornamental Plants of Ministry of Education, School of Landscape Architecture, Beijing Forestry University, Beijing 100083, China; yiyang921124@126.com (Y.Y.); 13683295193@163.com (M.S.); yuancunquan@163.com (C.Y.); hanyu19880514@126.com (Y.H.); zhengtangchun@126.com (T.Z.); chengtangren@163.com (T.C.); wangjia8248@163.com (J.W.); 2 Beijing Advanced Innovation Center for Tree Breeding by Molecular Design, Beijing Forestry University, Beijing 100083, China

**Keywords:** *Chrysanthemum morifolium*, WUS, CYC2, gynomonoecy, reproductive organ, flower symmetry

## Abstract

*Chrysanthemum morifolium* is a gynomonoecious plant that bears both female zygomorphic ray florets and bisexual actinomorphic disc florets in the inflorescence. This sexual system is quite prevalent in Asteraceae, but poorly understood. CYCLOIDEA (CYC) 2 subclade transcription factors, key regulators of flower symmetry and floret identity in Asteraceae, have also been speculated to function in reproductive organs and could be an entry point for studying gynomonoecy. However, the molecular mechanism is still unclear. On the other hand, the *Arabidopsis* WUSCHEL (WUS) transcription factor has been proven to play a vital role in the development of reproductive organs. Here, a *WUS* homologue (*CmWUS*) in *C. morifolium* was isolated and characterized. Overexpression of *CmWUS* in *A. thaliana* led to shorter siliques and fewer stamens, which was similar to *CYC2*-like genes reported before. In addition, both *CmWUS* and *CmCYC2* were highly expressed in flower buds during floral organ differentiation and in the reproductive organs at later development stages, indicating their involvement in the development of reproductive organs. Moreover, CmWUS could directly interact with CmCYC2d. Thus, our data suggest a collaboration between CmWUS and CmCYC2 in the regulation of reproductive organ development in chrysanthemum and will contribute to a further understanding of the gynomonoecious sexual system in Asteraceae.

## 1. Introduction

The inflorescence of *C. morifolium* (Asteraceae) is always comprised of two kinds of florets: the bilaterally symmetric female ray florets and radially symmetric bisexual disc florets [1]. Different sex expression and flower symmetry in ray and disc florets are significant features of chrysanthemum inflorescence. This gynomonoecious sexual system is quite prevailing in Asteraceae and has been considered to play a pivotal role in reducing herbivore damage and pollen-pistil interference, as well as in attracting pollinators [2,3]. However, the genetic mechanism of gynomonoecy is poorly understood. Interestingly, the connection between shifts in flower symmetry and the development of reproductive organs has been discovered and recorded in many species, including Asteraceae members [4,5,6], and the flower symmetry genes have been speculated to be involved in breeding system [6,7,8].

CYCLOIDEA (CYC) 2 subclade transcription factors, which belong to ECE-CYC/TB1 clade of plant-specific TCP family [9,10], have been proven to be essential for the regulation of flower symmetry in angiosperms [11] and inflorescence architecture in Asteraceae [6,12]. *CYC* of *Antirrhinum* was the first gene isolated in this subclade and is expressed in the dorsal domain of floral meristem from initiation and maintained throughout the differentiation of petals and stamens [13]. *CYC* promotes the growth of dorsal petals and arrests the development of dorsal stamen to form a staminode [13,14]. Gaudin et al. [15] speculated that *CYC* could directly or indirectly suppress the expression of *cyclinD3b* and other cell cycle genes in the staminode. Studies in *Opithandra* further indicated the negative effects of *OpdCYC* on *OpdcyclinD3* genes and the correlation between the expressions of *OpdCYC* and the abortion of both dorsal and ventral stamen [16]. On the other hand, Preston et al. [17] found that expression patterns of *CYC2*-like genes were not corelated with patterns of stamen arrest in *Veronica montana* and *Gratiola officinalis*. In contrast, in *Papaveracea*, *CYC2*-like genes promote stamen initiation and growth [18]. 

Previous studies have shown that *CYC2*-like genes in gerbera (*Gerbera hybrida*) are functionally redundant in regulating ray floret identity by promoting ligule growth and suppressing stamen development [19,20,21]. In addition to stamens, *CYC2*-like genes have also been speculated to have late functions in the development of ovaries and carpels in Asteraceae [6,12]. Both in gerbera and sunflower (*Helianthus annuus*), *CYC2*-like genes are highly expressed in ovary, stigma and style tissues [22]. Expression levels of *AcCYC2a* and *AcCYC2d* are also increased in the developing ovules of *Anacyclus clavatus* [23]. Moreover, constitutive expression of all the gerbera *CYC2*-like genes, except *GhCYC2*, in *A. thaliana* leads to shorter siliques with fewer seeds. In addition, stamen development is also severely disrupted in the transgenic lines ectopically expressing *GhCYC4* and *GhCYC7* [20]. Also, different from the empty achenes in the zygomorphic ray florets of wild type plants, actinomorphic ray florets can produce filled achenes through hand pollination in the *turf* mutant [24], which is caused by insertion of TEs in the TCP domain of *HaCYC2c* in sunflower [25,26,27]. Still, the molecular relationship between *CYC2*-like genes and the development of reproductive organs awaits more research to elaborate. 

In addition to *CYC2*-like genes, our previous comparative transcriptome analysis between ray and disc florets in *C. morifolium* [28] has predicted other candidate transcription factor genes during inflorescence development and organ determination for further studies. Among them, a *WUSCHEL*-like gene, which was highly expressed in the central disc florets, has attracted our attention. *WUSCHEL* (*WUS*) is a member of the WUSCHEL-RELATED HOMEOBOX (WOX) family [29] and takes part in several regulatory networks in shoot and floral meristems [30,31,32]. In *A. thaliana*, *WUS* is expressed in anther stomium cells during early stages and is required for anther development [33]. In ovules, *WUS* is confined to be expressed in the nucellus and is essential for the initiation of integument [34,35]. Reduced stamens and disappeared carpel in *wus* mutants of *Arabidopsis* also suggest crucial functions of *WUS* in the development of reproductive organs [36]. In *Cucumis sativus* (cucumber), CsWUS directly interacts with CsSPL, a vital factor in male and female fertility, and takes part in the regulatory network that controls the development of reproductive organs [37]. 

We have identified six *CmCYC2* genes in *C. morifolium* before, and they were also found to be strongly expressed in ray florets [38]. In contrast to *CmCYC2*, *CmWUS* was highly expressed in disc florets [28]. These two distinct expression patterns between ray and disc florets suggested their involvements in the development of inflorescence. In particular, whether *CmWUS* and *CmCYC2* are connected in reproductive organ development of chrysanthemum is an interesting problem worthy of study. Here, we isolated *CmWUS* and expressed it ectopically in *A. thaliana* for functional analysis. Additionally, expression patterns of *CmWUS* and *CmCYC2* during inflorescence development were compared. Furthermore, we performed yeast two-hybrid (Y2H) and bimolecular fluorescence complementation (BiFC) assays to determine protein-protein interactions between CmWUS and CmCYC2. These results show a direct interaction between CmWUS and CmCYC2 and may help to understand the genetic and molecular mechanisms of reproductive organ development in Asteraceae.

## 2. Results

### 2.1. Identification and Phylogenetic Analysis of CmWUS

To identify the function of *CmWUS* in reproductive organ development, we isolated the ORF of *CmWUS* (912 bp) from inflorescences of *C. morifolium* ‘Fen Ditan’. The encoded CmWUS protein (303 amino acids) was aligned with WUS-like sequences from other species. As shown in Supplementary Figure S1A, the WOX domain [29] was highly conserved. The signature motifs WUS-box and EAR-like motif [39] were also identified in CmWUS at the carboxyl terminus. A neighbor joining phylogenetic tree (Supplementary Figure S1B) was constructed based on the full length of amino acid sequences of 15 WOX family members from *A. thaliana* and WUS from other species. As described by Graaff, Laux and Rensing [29], these WOX members could be divided into three clades: the ancient clade, the intermediate clade and the WUS clade. The phylogenetic analysis confirmed that CmWUS belongs to WUS clade of WOX family and is closely related to WUS-like from other species of Asterceae: *H. annuus*, *L. sativa* and *C. cardunculus*. 

### 2.2. Overexpression of CmWUS in A. thaliana Inhibits the Development of Reproductive Organs and Affects Flower Symmetry

**Figure 1:**
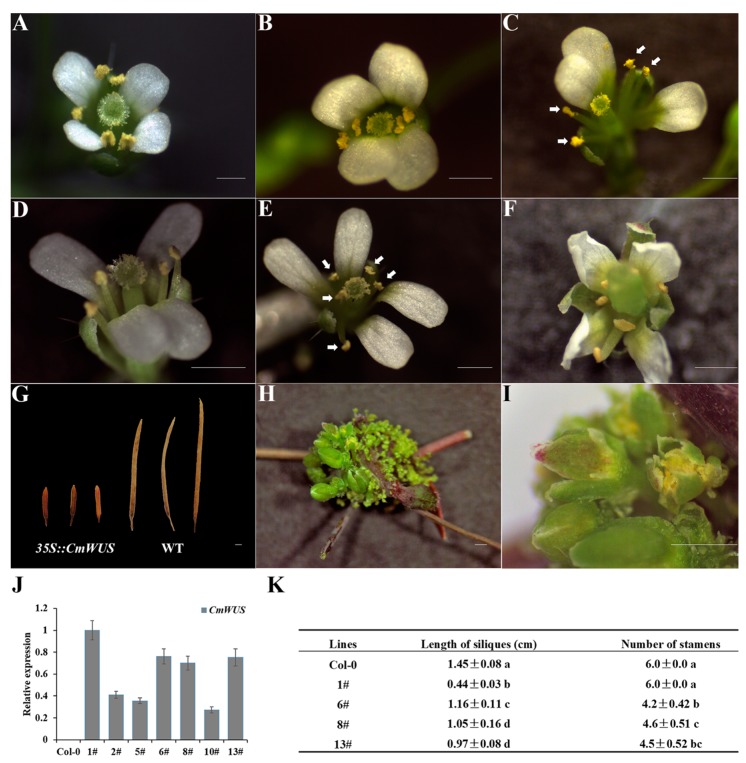
Ectopic expression of *CmWUS* in *A. thaliana* (Columbia). (**A**) Flower of wild type *A. thaliana* (Columbia). (**B–E**) Flower phenotypes in transgenic line 6, 8 and 13. Petals on both sides of the two lateral stamens were arranged close to each other and the development of the lateral stamens was also inhibited. The number of stamens were reduced to 4 (**B**,**C**) and 5 (**D**,**E**). Stamens are marked with white arrows. (**F**) Slightly curled petals at the edges of the flowers in transgenic line 1. (**G**) Siliques of transgenic line 1 (left) were much shorter than wild type (right). (**H**,**I**) Ectopic initiated flower buds on the surface of inflorescence stems in transgenic line 1. (**J**) qPCR detection of *CmWUS* transcripts in wild type (WT) and transgenic lines of *A. thaliana*. The endogenous *Arabidopsis ACTIN* was chosen as a housekeeper gene. (**K**) Statistics of silique length and stamen number in wild type and transgenic lines of *Arabidopsis*. Statistically significant differences are indicated with lowercase letters (Fisher’s LSD, *p* < 0.05). Bars = 1 mm.

The *CmWUS* ORF was overexpressed in *A. thaliana* (Columbia) for functional analysis during floral development. The transgenic lines in which *CmWUS* was highly expressed were confirmed by qPCR assay. Three *35S::CmWUS* lines (line 6, 8,13) with higher and consistent expression levels were selected for detailed analysis. The wild type *Arabidopsis* flowers are polysymmetric with four sepals, four petals, four medial and two lateral stamens and two fused carpels (Figure 1A). Meanwhile, in our transgenic lines, the flowers were changed into monosymmetric with one symmetry plane. The petals on both sides of the lateral stamens were arranged close to each other and the development of the lateral stamens was also inhibited (Figure 1B–E). As listed in Figure 1K, the number of stamens in three *35S::CmWUS* lines were reduced to 4 to 5. In addition, they produced shorter siliques than the wild type. In addition to these three transgenic lines, line 1 showed a stronger phenotype, with flower meristems that were ectopically initiated on the surface of inflorescence stems (Figure 1H,I), which was consistent with the phenotype of *Arabidopsis* overexpressing *AtWUS* [40,41]. Furthermore, petals were slightly curled at the edges (Figure 1F) and siliques were much shorter than wild type (Figure 1G) in line 1. 

### 2.3. High Expression of CmWUS and CmCYC2 in the Reproductive Organs of C. morifolium

**Figure 2:**
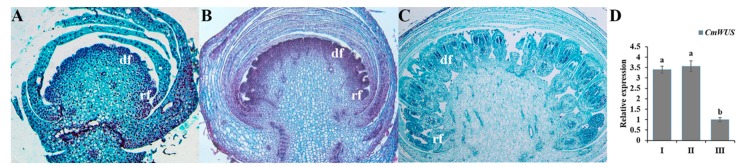
Expression patterns of *CmWUS* in flower buds of *C. morifolium* ‘Fen Ditan’ at early stages of inflorescence development. (**A–C**) Morphological characteristics of flower buds at three stages: I, initiation of floral primordia (**A**); II, differentiation of floral organs (**B**); and III, growth of floral organs (**C**) were analyzed at a histological level. Abbreviations: rf = ray florets, df = disc florets. (**D**) Expression levels of *CmWUS* in flower buds at stage I, II and III of inflorescence development. The expression levels are relative to the flower buds at stage III. Expression levels of *PP2Acs* are utilized for normalization. Error bars show the standard deviation of three biological replicates. Statistically significant differences are indicated with different lowercase letters (Fisher’s LSD, *p* < 0.05).

Three developmental phases of flower buds—initiation of floral primordia (I), differentiation of floral organs (II) and growth of floral organs (III) [38]—were selected (Figure 2A–C) to analyze the expression of *CmWUS* at early stages of inflorescence development in *C. morifolium.* As shown in Figure 2D, the expression level of *CmWUS* increased from stage I to stage II and then decreased to the lowest at stage III, which was similar to *CmCYC2* genes reported previously [38] and indicates their involvement in floral organ differentiation. 

**Figure 3:**
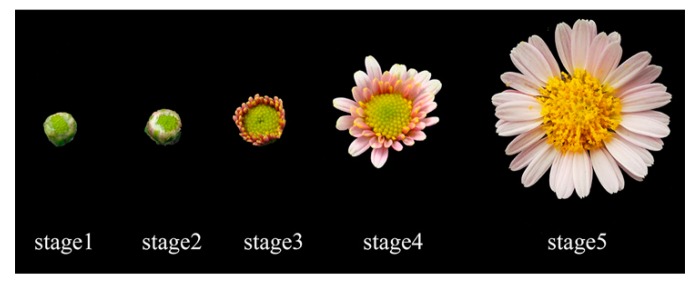
Inflorescence morphology of *C. morifolium* ‘Fen Ditan’ and five later stages of inflorescence development.

**Figure 4:**
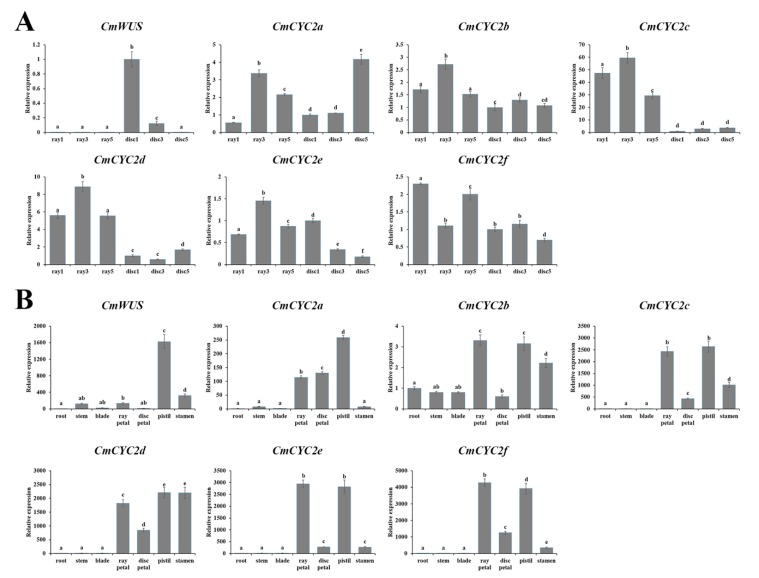
Comparative expression analysis of *CmWUS* and *CmCYC2* genes in *C. morifolium* ‘Fen Ditan’ at later stages of inflorescence development. (**A**) Gene expression patterns between ray and disc florets at later stages (stage 1, 3 and 5) of inflorescence development. The expression levels are relative to the disc florets at stage 1. (**B**) Relative expression levels of *CmWUS* and *CmCYC2* genes in different tissues of *C. morifolium* ‘Fen Ditan’. Tissues analyzed including: root, stem, blade, ray petal, disc petal, pistil (including stigma, style and ovary) and stamen. The expression levels are relative to the root sample. Expression levels of *PP2Acs* are utilized for normalization. Error bars show the standard deviation of three biological replicates. Statistically significant differences are indicated with different lowercase letters (Fisher’s LSD, *p* < 0.05).

qPCR assays were also performed to compare the expression patterns of *CmWUS* and *CmCYC2* at later stages of inflorescence development (Figure 3) between ray and disc florets. As shown in Figure 4A, *CmWUS* was expressed extremely highly in disc florets, especially at stage 1. The expression of *CmWUS* in ray florets was also detected, but was pretty weak compared to disc florets. Unlike *CmWUS*, *CmCYC2* genes, especially *CmCYC2c* and *CmCYC2d*, were expressed at relatively higher levels in ray florets than disc florets. To further explore the possible roles of *CmWUS* and *CmCYC2* genes, their expression levels in different tissues of *C. morifolium* ‘Fen Ditan’ at late development stages were studied. As shown in Figure 4B, *CmCYC2* and *CmWUS* were primarily expressed in floral organs and were strongly expressed in pistils (including ovary, style and stigma). *CmWUS* was also expressed in stamens, but the expression level was not as high as in pistils like *CmCYC2d*. *CmCYC2* genes were also expressed at high levels in petals, especially in ray petals, while *CmWUS* was not, which may explain the differences in expression levels of *CmWUS* and *CmCYC2* between ray and disc florets. Thus, we speculate that *CmWUS* and *CmCYC2* genes are all involved in the regulation of reproductive organ (especially the pistils) development.

### 2.4. Protein-Protein Interactions between CmWUS and CmCYC2

**Figure 5:**
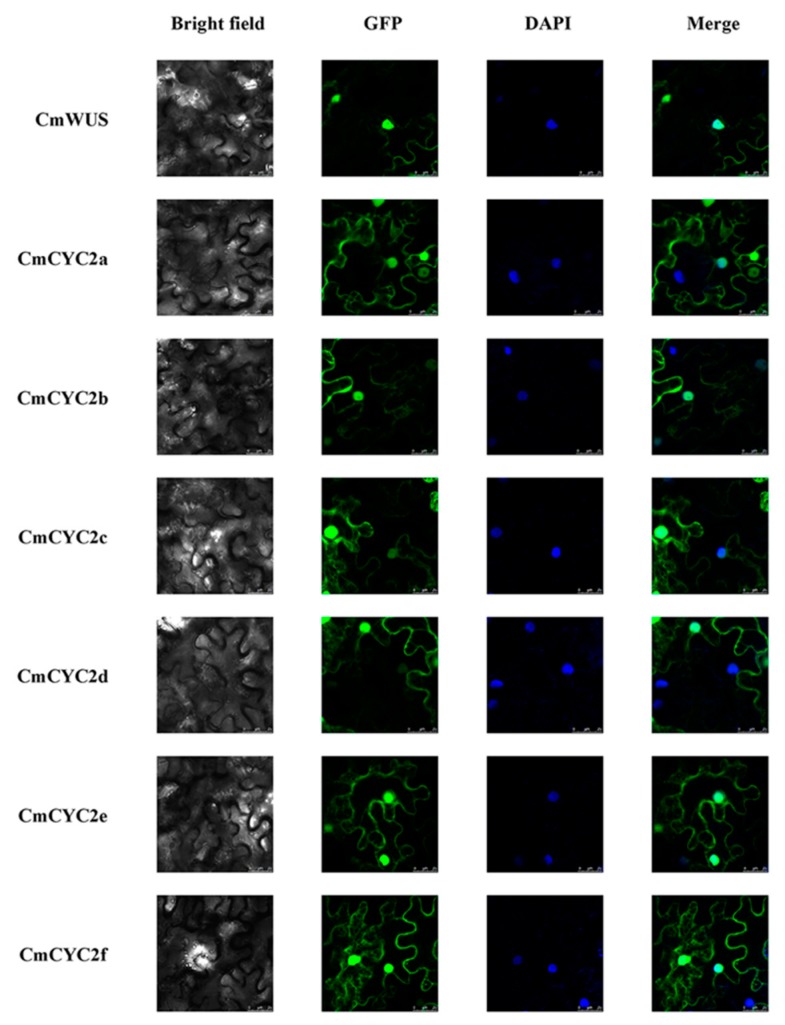
Subcellular localization of CmWUS and CmCYC2. pSuper1300-CmWUS and pSuper1300-CmCYC2 constructs were transiently transformed into the leaves of *Nicotiana benthamiana*. The fusion proteins (CmWUS-GFP and CmCYC2-GFP) were observed under the confocal laser scanning microscope. The merge pictures were made up of the GFP and DAPI pictures. The green and blue fluorescence show the position of proteins and nuclei, respectively. Bars = 25 μm.

**Figure 6:**
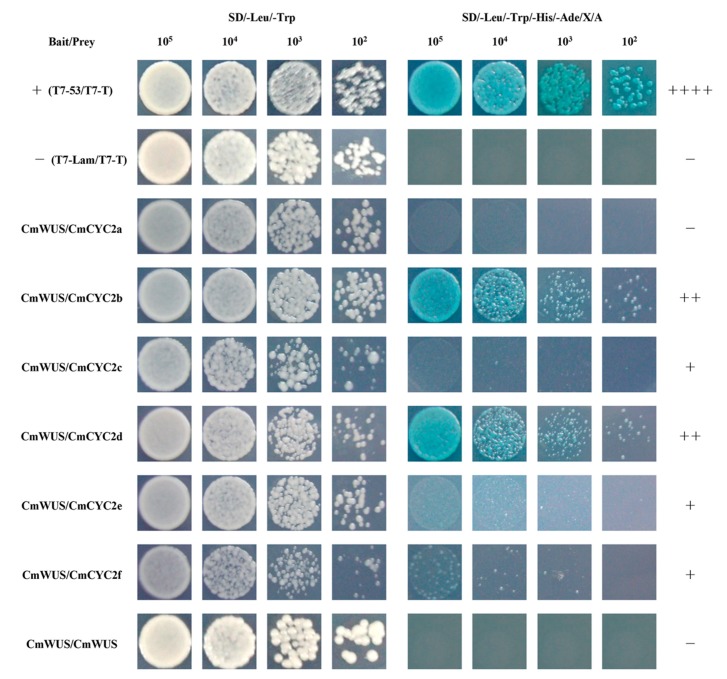
Yeast two-hybrid (Y2H) analysis of protein-protein interactions between CmWUS and CmCYC2. Clones containing each combination of bait and prey vectors were cultured on both nonselective media (SD/-Trp/-Leu) and selective media (SD/-Leu/-Trp/-His/-Ade/X/A). T7-53/T7-T and T7-Lam/T7-T are the positive and negative control. “+” represents the intensity of the interaction and “-” means no interaction.

**Figure 7:**
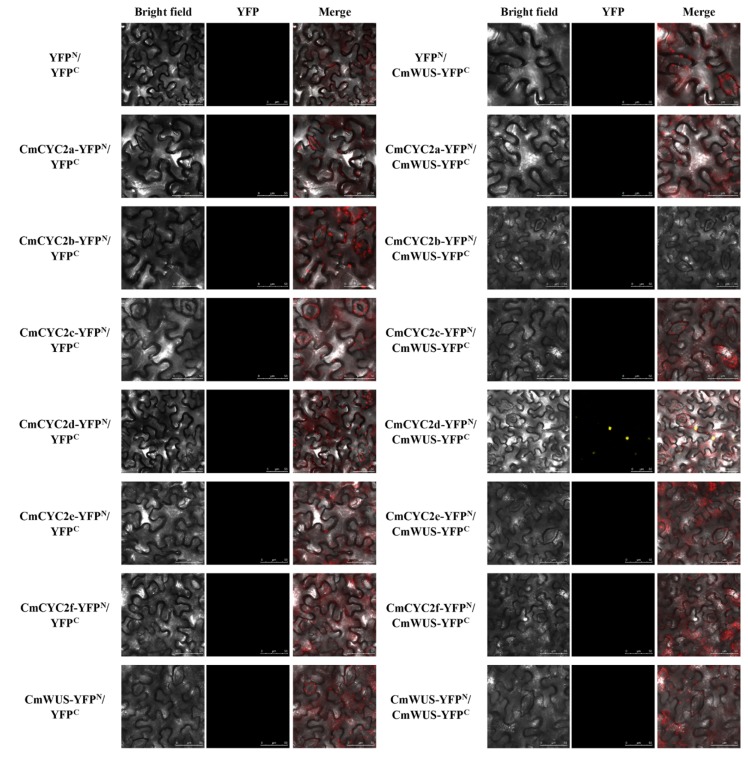
Bimolecular fluorescence complementation (BiFC) analysis of the interactions between CmWUS and CmCYC2 proteins in the epidermal cells of *N. benthamiana* leaves. CmCYC2 and CmWUS were fused to the N-terminal and C-terminal fragment of pCambia1300-YFP respectively and then co-transformed into *N. benthamiana* leaf cells. The confocal laser scanning microscope was used for visualizing. The yellow fluorescence shows the position of protein. Bars = 50 μm.

Since *CmWUS* and *CmCYC2* were both highly expressed in the reproductive organs, we further examined the interactions between CmWUS and CmCYC2 to reveal their relationship. The GFP and DAPI fluorescence indicated that CmWUS and CmCYC2 were mainly localized to the cell nucleus (Figure 5). In yeast two-hybrid (Y2H) assays, CmWUS had no autoactivation activity and was used as a bait. The results are shown in Figure 6. CmWUS could not form a homodimer, which was the opposite to the results in *Arabidopsis*, and this may be caused by the differences in the homodimerization interacting amino acids at the central part of the CmWUS sequence (Supplementary Figure S1A) [42]. Furthermore, CmWUS could dimerize with CmCYC2b and CmCYC2d, and the interactions with CmCYC2c, CmCYC2e and CmCYC2f were quite weak. Bimolecular fluorescence complementation (BiFC) assays were performed to provide further evidence for the interactions. There was no interaction in YFP^N^/YFP^C^, CmCYC2-YFP^N^/YFP^C^, CmWUS-YFP^N^/YFP^C^ or YFP^N^/CmWUS-YFP^C^ combinations. As shown in Figure 7, only in the combination of CmCYC2d-YFP^N^/CmWUS-YFP^C^, YFP fluorescence was detected. Taken together, CmWUS could directly interact with CmCYC2d, and the CmWUS-CmCYC2d complex is localized to the cell nucleus.

## 3. Discussion

### 3.1. Ectopic Expression of CmWUS in A. thaliana Indicates Possible Conserved Functions in Floral Meristems 

Bifunctional transcription factor WUS plays a vital role in the stem cell maintenance of shoot and floral meristems and has been proven to be sufficient for the meristem reestablishment in the inflorescence stem [39,40,41]. To elucidate the functions of *CmWUS*, we first analyzed the sequence in detail. The WUS-box motif, which was elementary for WUS function in both shoot and floral meristems [39], was highly conserved. Also, the transcriptional repression related EAR motif [43] was identified at the carboxyl terminus. We further explored the function of *CmWUS* during flower development through overexpression in *A. thaliana*. In our transgenic line 1, clustered flower buds were ectopically initiated on the inflorescence stems. This phenotype was consistent with *sef*, a gain-of-function mutant caused by the overexpression of endogenous *WUS* [41]. Therefore, we speculated that *CmWUS* may retain conserved functions in floral meristems. In *sef* mutant, the floral identity gene *LFAFY (LFY)* was also activated [41] and it could cooperate with *WUS* to activate *AGAMOUS (AG)*, a MADS-box gene which specifies the identity of carpel and stamen [30,39,44]. This *WUS/LFY-AG* regulatory loop could be a possible explanation of the ectopic floral buds [40,41]. 

Another noteworthy phenotype in transgenic line 1 was the curled petals, indicating more active cell proliferation in abaxial side. *WOX1* and *WOX3*, which belong to WUS clade of WOX family [29], have been reported to regulate leaf and floral organ development and affect the abaxial-adaxial balance [45,46]. Thus, *CmWUS* may also be involved in petal morphogenesis through the regulation of abaxial-adaxial patterning. However, this still requires more research to elucidate. 

### 3.2. Proposed Interaction between CmWUS and CmCYC2 in Regulating Reproductive Organ Development

Changes in the number of stamens always come after the shifts in flower symmetry, and it has been reported in Asteraceae that mutations of floret symmetry could affect the development of stamens and carpels [6,47]. *CYC2*-like genes, key factors of flower symmetry, are vital in determining floret identity and regulating floral organ development in Asteraceae [12]. In the transgenic *Arabidopsis* lines with constitutive expression of gerbera *CYC2*-like genes, the siliques were shorter than wild type. Moreover, overexpression of *GhCYC4* and *GhCYC7* could disrupt the development of petals and stamens and carpels were unable to produce normal siliques [20]. In this study, *35S::CmWUS* lines also produced shorter siliques and fewer stamens with variations in flower symmetry. In addition, the transcriptional level of *CmWUS* and *CmCYC2* genes during inflorescence development were compared in chrysanthemum. All the genes were highly expressed at the early stages of flower bud differentiation [38] and may be involved in floral organ development. At later stages, tissue-specific expression analysis revealed that they were all highly expressed in reproductive organs. In general, based on the transgenic *Arabidopsis* phenotypes and gene expression patterns, we conclude that *CmWUS* and *CmCYC2* genes may play an important role in the development of reproductive organs in chrysanthemum. Furthermore, Y2H and BiFC analyses indicated that CmWUS directly interact with CmCYC2d, an ortholog of GhCYC3 that has been proven to suppress stamen development in gerbera [20,38]. Hence, CmWUS and CmCYC2d may act together to affect the development of reproductive organs. This may further explain the mechanism of *CYC2*-like genes in the regulation of reproductive organ development. In addition, previous studies of CYC2-like proteins in gerbera and sunflower have shown redundant functions and higher capacity to form dimers within CYC2 subclade [12,19,20,22,26]. Thus, CmCYC2d could be the mediator between CmWUS and CmCYC2 and a complex regulatory network involving CmWUS and CmCYC2 subclade may exist in regulating reproductive organ development in chrysanthemum.

### 3.3. WUS Can Be a Bridge to Connect MADS-box and ECE (CYC/TB1)

It has been speculated that the flower morphology-related ECE and MADS-box genes may be closely linked [6,9,12,48]. In *Antirrhinum*, B-class MADS-box gene *DEF* and C-class gene *PLENA* are suggested to be required in the maintenance of *CYC* in whorl 2 and whorl 3, respectively [49]. *CYC2*-like genes are also indicated to be involved in regulating sepal identity by suppressing B-class genes in *Cysticapnos* [18]. In the *mtaga mtagb* double mutant of *Medicago truncatula*, the abnormal petals are related to the upregulation of *CYC2*-like genes [50]. Also, *GhSOC1* is thought to function upstream of CYC2 subclade genes in *Gerbera* [12,51]. However, the regulatory connections between MADS-box and ECE genes still remain to be illustrated. On the other hand, WUS acts as an activator in regulating the expression of C-class MADS-box gene *AG* in floral patterning and *AG* represses *WUS* directly or indirectly through activation of *KNUCKLES* at later stages of floral development in turn [39,44,52]. Furthermore, an A-class gene, *APETALA2 (AP2)*, antagonizes *AG* through promoting the expression of *WUS* in the floral meristem [53]. In this study, *CmWUS* and *CmCYC2* were found to be highly expressed in the reproductive organs of chrysanthemum and CmWUS could directly interact with CmCYC2d. A connection between WUS and ECE was established. Taken together, WUS, ECE and MADS-box may be linked together during floral development and WUS acts as the adaptor to connect MADS-box and ECE.

In conclusion, this study characterized a *WUS*-like gene, *CmWUS*, in *C. morifolium* and revealed a remarkable link between CmWUS and CmCYC2 subclade. Since the significant function of CmWUS in reproductive organ development, our findings will help fill in the missing link of CmCYC2 in regulating the development of reproductive organs, especially in pistils, and contribute to a further understanding of the molecular mechanisms of gynomonoecy in Asteraceae.

## 4. Materials and Methods

### 4.1. Plant Materials and Growth Condition

*C. morifolium* ‘Fen ditan’ (Figure 3) and *A. thaliana* were cultivated in a greenhouse of Beijing Forestry University, China. They were grown under photoperiods of 8 h light (24 ℃)/16 h dark (20 ℃) and 16 h light (22 ℃)/8 h dark (19 ℃), respectively.

### 4.2. Gene Cloning

Total RNA was extracted from the inflorescences of *C. morifolium* ‘Fen Ditan’ with Plant RNA Kit (Omega, Norcross, GA, USA), and then used as template to synthesize first strand of cDNA with TransScript One-Step gDNA Removal and cDNA Synthesis SuperMix (Transgen, Beijing, China). Partial sequence of *CmWUS* in chrysanthemum was retrieved from our previously published RNAseq data [28]. SMARTerTM RACE 5′/3′ Kit (Clontech, Mountain View, CA, USA) was used for 5′ and 3′ RACE. 5′-GSP and 3′-GSP (Supplementary Table S1), gene-specific primers for RACE, were designed according to the instructions. Based on the 5′- and 3′-ends, *CmWUS*-F1 and *CmWUS*-R1 (Supplementary Table S1) were designed to amplify the open reading frame (ORF) sequence of *CmWUS*. Six *CmCYC2* genes (GenBank ID: *CmCYC2a*, KU595430.1; *CmCYC2b*, KU595431.1; *CmCYC2c*, KU595428.1; *CmCYC2d*, KU595426.1; *CmCYC2e*, KU595427.1; *CmCYC2f*, KU595429.1) were amplified with primers reported before [38]. All the PCR products were cloned into pCloneEZ-Blunt TOPO vectors (Taihe, Beijing, China), transformed into *Escherichia coli* DH5α cells (Tiangen, Beijing, China) and sequenced by Taihe (Beijing, China). The coding sequence of *CmWUS* (GenBank accession number: MK124768) has been uploaded to the NCBI database.

### 4.3. Bioinformatics Analysis

ClustalX software was used to perform alignment of multiple sequences, including CmWUS and WUS-like sequences from other species. GeneDoc software was used to edit the alignment. A phylogenetic tree was constructed by MEGA 7 based on the neighbor-joining method with 1000 bootstrap replicates, using the full length of the amino acid sequences of WUS homologs from various species and 15 WOX family members from *A. thaliana*. The accession numbers of sequences used here were as follows: AtWUS, *A. thaliana*, NM_127349.4; AtWOX1, AY251394.1; AtWOX2, NM_125325.3; AtWOX3, NM_128422.3; AtWOX4, FJ440850.1; AtWOX5, AY251398.1; AtWOX6, AY251399.2; AtWOX7, NM_120659.2; AtWOX8, AY251400.1; AtWOX9, AY251401.1; AtWOX10, NM_101923.1; AtWOX11, AY251402.1; AtWOX12, AY251403.1; AtWOX13, AY251404.1; AtWOX14, NM_101922.3; AmWUS, *Antirrhinum majus*, AAO23113.1; BnWUS, *Brassica napus*, XM_013803833.2; CcWUS, *Cynara cardunculus* var. *scolymus*, XM_025106474.1; CsWUS, *Citrus sinensis*, NM_001288918.1; GmWUS, *Glycine max*, XP_003517180.2; HaWUS, *Helianthus annuus*, HE616565.1; LsWUS, *Lactuca sativa*, XM_023909093.1; MtWUS, *Medicago truncatula*, XP_003612158.1; NtWUS, *Nicotiana tabacum*, XM_016619508.1; SlWUS, *Solanum lycopersicum*, ADZ13564.1; StWUS, *Solanum tuberosum*, XP_006340731.1; VvWUS, *Vitis vinifera*, XM_002266287.3.

### 4.4. Overexpression of CmWUS in A. thaliana

*CmWUS* was amplified using primers *CmWUS*-F2 and *CmWUS*-R2 (Supplementary Table S1) and subcloned into *Nco*I/*Bst*EII-cleaved pCambia1304 vector under the CaMV35S promoter using In-Fusion^®^ HD Cloning Kit System (Clontech, Mountain View, CA, USA). The resulting pCambia1304-*CmWUS* vector was transformed into *A. thaliana* (Columbia) via *Agrobacteriaum tumefaciens* GV3101with the floral dip method [54]. The seeds were selected on MS medium containing hygromycin B (50 mg/L; Roche, Basel, Switzerland). qRT-PCR was performed using young leaves to confirm positive lines with primers *CmWUS*-F3/R3 and *AtACTIN*-F/R (Supplementary Table S1). Three independent homozygous T_3_ lines with higher and consistent expression levels were selected for floral phenotype analysis. Forty flowers were analyzed and the significant differences were determined according to Fisher’s LSD (*p* < 0.05) with SPSS 20.0.

### 4.5. Microscope Observations

The floral buds of *C. morifolium* ‘Fen Ditan’ at different stages were fixed in FAA (50% ethanol: acetic acid: formaldehyde = 90:5:5, v/v), dehydrated with a graded ethanol series (50%–100%) and then transferred into xylene (100%). All the samples were embedded in paraffin and cut into 8 μm sections using a microtome (Leica, Wetzlar, Germany). After that, paraffin was removed from the sections with xylene, and then safranin (1%) and fast green (0.5%) were used for histological staining. All the sections were examined and photographed under a light microscope (Zeiss, Jena, Germany) after sealed with neutral gum.

### 4.6. Gene Expression Analysis in C. morifolium

Floral buds of *C. morifolium* ‘Fen Ditan’ at different stages were collected for analysis of gene expression patterns. To compare expression patterns of *CmWUS* and *CmCYC2* genes at later stages of inflorescence development between ray and disc florets, samples were pooled from the flower heads of *C. morifolium* ‘Fen Ditan’ at different stages (Figure 4). To analyze tissue-specific expression of *CmWUS* and *CmCYC2* genes, vegetative and reproductive tissues were collected from the inflorescences of *C. morifolium* ‘Fen Ditan’ at stage 4 and 5 of inflorescence development (Figure 4). Particularly, pistil samples were dissected from both ray and disc florets, while stamen samples were pooled from disc florets only. Total RNA was extracted as described above and PrimeScriptTM RT reagent Kit (Perfect Real Time; TaKaRa, Shiga, Japan) was used to synthesize the first strand of cDNA. Quantitative real-time PCR experiments were performed using the PikoReal real-time PCR system (Thermo Fisher Scientific, Waltham, MA, USA) with a 10 μL mix of SYBR Premix ExTaq II (5 μL; Takara, Shiga, Japan), forward and reverse primers (10 μM, 0.5 μL each), cDNA (2 μL) and sterile distilled water (2 μL). The qPCR primers of *CmCYC2* genes and the reference gene *PP2Acs* were reported before [28,38,55]. *CmWUS*-F3/R3 (Supplementary Table S1) was used as qPCR primer of *CmWUS*. Three biological replicates were conducted with three technical replicates each. 2^–ΔΔCt^ method [56] was used to calculate the relative expression levels. 

### 4.7. Subcellular Localization

*CmWUS* and *CmCYC2* genes were amplified and subcloned into *Sall*/*Spel*-cleaved pSuper1300-*GFP* vectors to generate the transformation plasmids *35S::CmWUS::GFP* and *35S::CmCYC2::GFP*. The plasmids were transformed into *A. tumefaciens* and injected into the leaves of *Nicotiana benthamiana* following the procedure reported before [57]. TCS SP8 (Leica, Wetzlar, Germany) confocal laser scanning microscope was used to assess subcellular localization at 488 and 408 nm for GFP and DAPI fluorescence, respectively. Primers used for subcellular localization are listed in Supplementary Table S1.

### 4.8. Y2H Assay

Matchmaker Gold Yeast Two-Hybrid System (Clontech, Mountain View, CA, USA) was used to carry out Y2H assays. *CmCYC2* and *CmWUS* were amplified and subcloned into the pGADT7 (prey) and pGBKT7 (bait) vectors. The reconstructed pGADT7 and pGBKT7 vectors were transformed into Y187 and Y2H gold yeast strains and cultured on SD/-Leu and SD/-Trp plates, respectively. If the colonies containing bait vector are significantly smaller than colonies containing the empty pGBKT7 vector on SD/-Trp plates, then the bait is toxic to the yeast cells. To test the bait for autoactivation, Y2H gold yeast cells containing pGBKT7-*CmWUS* vector were cultured on SD/-Trp, SD/-Trp/X-α-Gal SD/-Trp/X-α-Gal/Aureobasidin A (AbA) and SD–Trp/–His/–Ade plates. If the colonies grow on both SD/-Trp and SD/-Trp/X-α-Gal plates, but not on SD/-Trp/X-α-Gal/AbA and SD–Trp/–His/–Ade plates, then the bait cannot autoactivate the AbA^r^ and His3/Ade2 reporter. After the testing of toxicity and autoactivation, diploid mating was conducted as described previously [58], and the transformed colonies were cultured on SD/-Trp/-Leu and SD/-Leu/-Trp/-His/-Ade/X-α-Gal/AbA (SD/-Leu/-Trp/-His/-Ade/X/A) plates to test for possible interactions. Y2H screenings were performed in triplicate. Primers used for Y2H assays are listed in Supplementary Table S1.

### 4.9. BiFC Assay

*CmCYC2* and *CmWUS* genes were amplified and subcloned into the pCambia1300-YFP^N^ and pCambia1300-YFP^C^ vectors. Co-expression was conducted in the leaves of tobacco (*N. benthamiana*) as described in Subcellular Localization. TCS SP8 (Leica, Wetzlar, Germany) confocal laser scanning microscope was used to detect YFP fluorescence at 514 nm. Primers used for BiFC assays are listed in Supplemental Table S1.

## Supplementary Materials

Supplementary materials can be found at https://www.mdpi.com/1422-0067/20/6/1276/s1. Figure S1: Alignment and phylogenetic analysis of CmWUS; Table S1: Primers used.
