# Supplementary material for: Interactions between WUSCHEL- and CYC2-like Transcription Factors in Regulating the Development of Reproductive Organs in Chrysanthemum morifolium
Source: Int J Mol Sci. 2019 Mar 14;20(6):1276. doi: 10.3390/ijms20061276 (PMC6471657; doi:10.3390/ijms20061276)
Supplement: Supplementary file 1 [file ijms-20-01276-s001.zip › Supplementary Figure S1.docx]

Supplementary Material

Interactions between WUSCHEL- and CYC2-like Transcription Factors in Regulating the Development of Reproductive Organs in Chrysanthemum morifolium

Yi Yang , Ming Sun , Cunquan Yuan , Yu Han , Tangchun Zheng , Jia Wang , Tangren Cheng and Qixiang Zhang*

* Correspondence: Qixiang Zhang: [zqxbjfu@126.com](mailto:zqxbjfu@126.com)

| 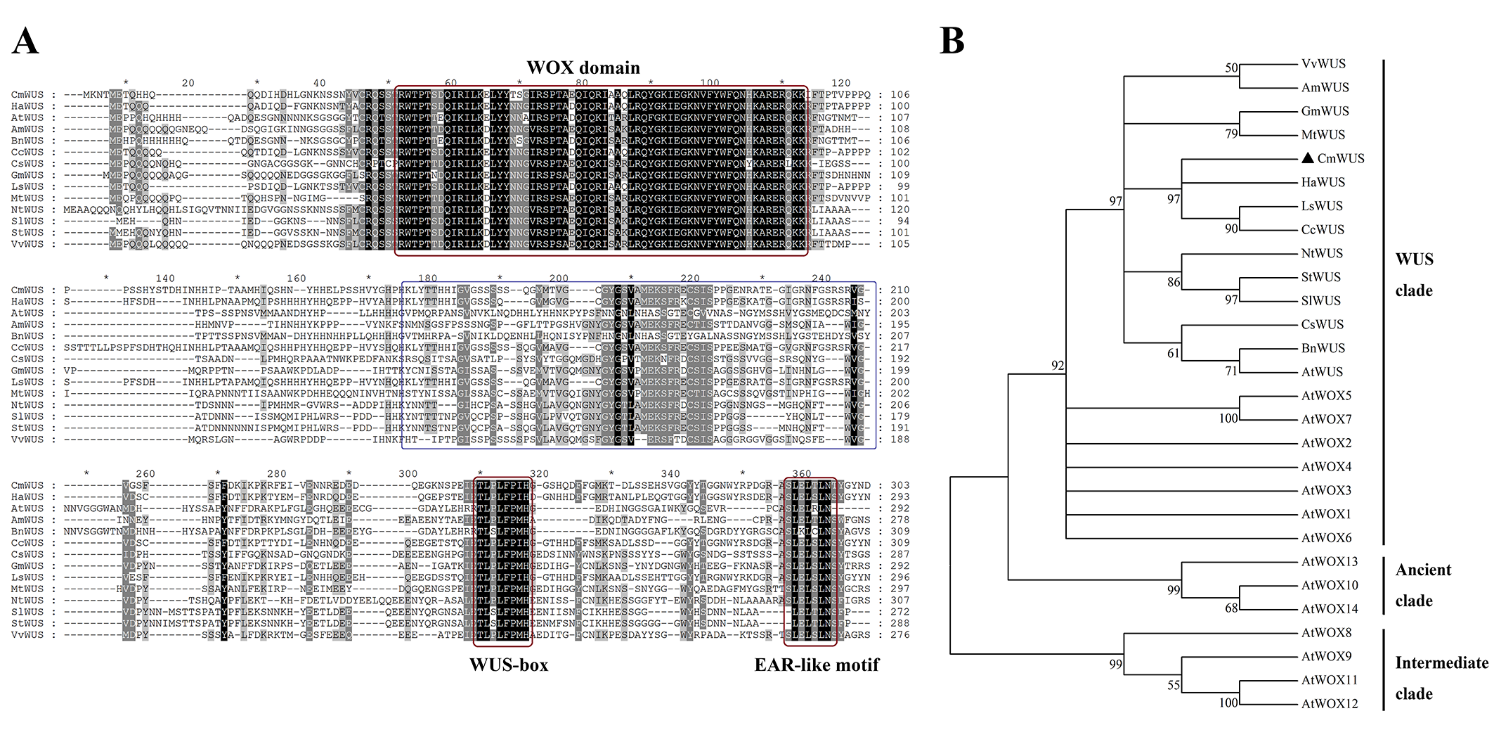 |
| --- |

**Supplementary Figure S1.** Alignment and phylogenetic analysis of CmWUS. **(A)** Multiple alignment of amino acid sequences of CmWUS and WUS-like sequences from other species. The WOX domain, WUS-box and EAR-like motif are boxed in red. The homodimerization interacting amino acids at the central part are boxed in blue. **(B)** Phylogenetic analysis of WUS homologs from various species and 15 WOX family members from *A. thaliana* using neighbor-joining method with 1000 bootstrap replicates. CmWUS belongs to WUS clade of WOX family. Accession numbers of the sequences used here are listed in ‘Materials and Methods’.
