# Supplementary material for: Interactions between WUSCHEL- and CYC2-like Transcription Factors in Regulating the Development of Reproductive Organs in Chrysanthemum morifolium
Source: Int J Mol Sci. 2019 Mar 14;20(6):1276. doi: 10.3390/ijms20061276 (PMC6471657; doi:10.3390/ijms20061276)
Supplement: Supplementary file 1 [file ijms-20-01276-s001.zip › Supplementary Table S1.docx]

Supplementary Material

Interactions between WUSCHEL- and CYC2-like Transcription Factors in Regulating the Development of Reproductive Organs in Chrysanthemum morifolium

Yi Yang , Ming Sun , Cunquan Yuan , Yu Han , Tangchun Zheng , Jia Wang , Tangren Cheng and Qixiang Zhang*

* Correspondence: Qixiang Zhang: [zqxbjfu@126.com](mailto:zqxbjfu@126.com)

**Supplementary Table S1. Primers used**

| Primer names | DNA sequence (5’-3’) | Experiment |
| --- | --- | --- |
| 5’-GSP | ATCACTTGTGGGAGTCCACCTGG | 5’-RACE of *CmWUS* |
| 3’-GSP | CGCGTGTGGGAGTCGGTTCATTT | 3’- RACE of *CmWUS* |
| *CmWUS*-F1 | ATGAAAAACACAATGGAAACACAAC | *CmWUS* ORF sequence  cloning |
| *CmWUS*-R1 | TCAGTCGTTATATCCATATGTGTTG |  |
| *CmWUS*-F2 | GGACTCTTGACCATGGTTATGAAAAACACAATGGAAACACAAC | Overexpression of *CmWUS* in  *Arabidopsis thaliana* |
| *CmWUS*-R2 | ATTCGAGCTGGTCACCTCAGTCGTTATATCCATATGTGTTG |  |
| *CmWUS*-F3 | ACACCAGTGGAATCAGATCACC | qPCR |
| *CmWUS*-R3 | GCCTTCAATCTTGCCGTACT |  |
| *AtACTIN*-F | GGTATGGGTCAGAAAGATGCT |  |
| *AtACTIN*-R | CGTTGTAGAAAGTGTGATGCC |  |
| *CmWUS-*F4 | AGGGGCCCGGGGTCGACATGAAAAACACAATGGAAACACAAC | Subcellular Localization |
| *CmWUS-*R4 | GGTACCGGATCCACTAGTGTCGTTATATCCATATGTGTTG |  |
| *CmCYC2a-*F1 | AGGGGCCCGGGGTCGACATGTTTAATTCTTCAAATCC |  |
| *CmCYC2a-*R1 | GGTACCGGATCCACTAGTATCCTCTTTCGATTCAA |  |
| *CmCYC2b-*F1 | AGGGGCCCGGGGTCGACATGTTTTCATCAAATCTTTTTCCA |  |
| *CmCYC2b-*R1 | GGTACCGGATCCACTAGTAGTTCTTTGCTGTTCCA |  |
| *CmCYC2c-*F1 | AGGGGCCCGGGGTCGACATGTTTTCCTCAAACCCCTT |  |
| *CmCYC2c-*R1 | GGTACCGGATCCACTAGTCAACATCAGTCCAGGTC |  |
| *CmCYC2d-*F1 | AGGGGCCCGGGGTCGACATGTTTTCCTCGAACCCTT |  |
| *CmCYC2d-*R1 | GGTACCGGATCCACTAGTGTGTAAATTTAGGAAACTTGTG |  |
| *CmCYC2e-*F1 | AGGGGCCCGGGGTCGACATGTTTTCCACAAATCCCTATTCAC |  |
| *CmCYC2e-*R1 | GGTACCGGATCCACTAGTAATAGGGTGACGGTCGC |  |
| *CmCYC2f-*F1 | AGGGGCCCGGGGTCGACATGATGTTTTCCCCAAACCA |  |
| *CmCYC2f-*R1 | GGTACCGGATCCACTAGTCTTGTTTAAACAAAGACCTTGA |  |
| *CmWUS-*F5 | CATGGAGGCCGAATTCATGAAAAACACAATGGAAACACAAC | Y2H assay  pGBKT7 construction |
| *CmWUS-*R5 | GCAGGTCGACGGATCCTCAGTCGTTATATCCATATGTGTTG |  |
| *CmWUS-*F6 | GGAGGCCAGTGAATTCATGAAAAACACAATGGAAACACAAC | Y2H assay  pGADT7 construction |
| *CmWUS-*R6 | CGAGCTCGATGGATCCTCAGTCGTTATATCCATATGTGTTG |  |
| *CmCYC2a-*F2 | GGAGGCCAGTGAATTCATGTTTAATTCTTCAAATCC |  |
| *CmCYC2a-*R2 | CGAGCTCGATGGATCCTTAATCCTCTTTCGATTCAA |  |
| *CmCYC2b-*F2 | GGAGGCCAGTGAATTCATGTTTTCATCAAATCTTTTTCCA |  |
| *CmCYC2b-*R2 | CGAGCTCGATGGATCCTTAAGTTCTTTGCTGTTCCA |  |
| *CmCYC2c-*F2 | GGAGGCCAGTGAATTCATGTTTTCCTCAAACCCCTT |  |
| *CmCYC2c-*R2 | CGAGCTCGATGGATCCTTACAACATCAGTCCAGGTC |  |
| *CmCYC2d-*F2 | GGAGGCCAGTGAATTCATGTTTTCCTCGAACCCTT |  |
| *CmCYC2d-*R2 | CGAGCTCGATGGATCCCTAGTGTAAATTTAGGAAACTTGTG |  |
| *CmCYC2e-*F2 | GGAGGCCAGTGAATTCATGTTTTCCACAAATCCCTATTCAC |  |
| *CmCYC2e-*R2 | CGAGCTCGATGGATCCCTAAATAGGGTGACGGTCGC |  |
| *CmCYC2f-*F2 | GGAGGCCAGTGAATTCATGATGTTTTCCCCAAACCA |  |
| *CmCYC2f-*R2 | CGAGCTCGATGGATCCCTACTTGTTTAAACAAAGACCTTGA |  |
| *CmWUS-*F7 | TGCAGGGAGGAGGATCCATGAAAAACACAATGGAAACACAAC | BiFC assay |
| *CmWUS-*R7 | CGGTGCACTAGTGTCGACGTCGTTATATCCATATGTGTTG |  |
| *CmCYC2a-*F3 | TGCAGGGAGGAGGATCCATGTTTAATTCTTCAAATCC |  |
| *CmCYC2a-*R3 | CGGTGCACTAGTGTCGACATCCTCTTTCGATTCAA |  |
| *CmCYC2b-*F3 | TGCAGGGAGGAGGATCCATGTTTTCATCAAATCTTTTTCCA |  |
| *CmCYC2b-*R3 | CGGTGCACTAGTGTCGACAGTTCTTTGCTGTTCCA |  |
| *CmCYC2c-*F3 | TGCAGGGAGGAGGATCCATGTTTTCCTCAAACCCCTT |  |
| *CmCYC2c-*R3 | CGGTGCACTAGTGTCGACCAACATCAGTCCAGGTC |  |
| *CmCYC2d-*F3 | TGCAGGGAGGAGGATCCATGTTTTCCTCGAACCCTT |  |
| *CmCYC2d-*R3 | CGGTGCACTAGTGTCGACGTGTAAATTTAGGAAACTTGTG |  |
| *CmCYC2e-*F3 | TGCAGGGAGGAGGATCCATGTTTTCCACAAATCCCTATTCAC |  |
| *CmCYC2e-*R3 | CGGTGCACTAGTGTCGACAATAGGGTGACGGTCGC |  |
| *CmCYC2f-*F3 | TGCAGGGAGGAGGATCCATGATGTTTTCCCCAAACCA |  |
| *CmCYC2f-*R3 | CGGTGCACTAGTGTCGACCTTGTTTAAACAAAGACCTTGA |  |
